# Secure Multi-User Near-Field Transmission with Movable Antennas and a Stacked Intelligent Metasurface

**DOI:** 10.3390/e28101153

**Published:** 2026-10-20

**Authors:** Yu Zhang, Junyu Tan, Xinyue Hu, Juanjuan Zhang, Xingyu Zhao, Yaxuan Fan

**Affiliations:** School of Electronics and Information Engineering, Anhui University, Hefei 230601, China; zhyu@ahu.edu.cn (Y.Z.);

**Keywords:** movable antenna, stacked intelligent metasurface, near-field communication, physical-layer security, secrecy sum rate, alternating optimization

## Abstract

This paper studies secure multi-user near-field transmission assisted by movable antennas (MAs) and a stacked intelligent metasurface (SIM) in the presence of multiple eavesdroppers. Each transmit antenna moves continuously along a common rail subject to aperture and minimum-spacing constraints, so its position independently controls one column of the near-field MA–SIM channel. We formulate the maximization of the secrecy sum rate against the strongest eavesdropper for each stream by jointly optimizing the transmit beamformers, MA positions, and SIM phase shifts. An alternating optimization algorithm combines a weighted minimum mean-square error (WMMSE) beamforming update, a layer-by-layer SIM phase update, and a successive convex approximation (SCA) update of all MA positions. Numerical results demonstrate that the proposed joint design outperforms the benchmarks, achieving secrecy sum rates more than 20% higher than those of the fixed uniform linear array (ULA) + SIM and MA-only schemes. The same-angle simulation further shows that spherical-wave range dependence enables positive secrecy rates when a plane-wave model yields zero, highlighting the role of near-field range discrimination.

## 1. Introduction

Over the past decades, physical-layer security (PLS) in far-field wireless communications has garnered substantial research interest. It leverages the inherent randomness of wireless propagation channels to endow legitimate users with a rate advantage over eavesdroppers, obviating the need for complex upper-layer secret keys [1]. To meet the high-data-rate demands of 6G applications, extremely large-scale multiple-input multiple-output (XL-MIMO) has emerged as a prominent solution, deploying far more antennas than conventional massive MIMO to enhance spectral efficiency and spatial resolution [2]. Nevertheless, this enlarged aperture inevitably pushes the communication regime into the near field, where the widely used far-field planar-wave model becomes inaccurate due to non-negligible phase discrepancies. It is therefore necessary to investigate PLS in the near field.

The spherical wavefront in the near field introduces an additional range dimension, allowing confidential signals to be focused at a desired location rather than merely steered toward a desired angle [3,4]. This location-dependent focusing capability offers inherent advantages for near-field PLS. A hybrid-beamforming framework demonstrated that near-field secure transmission can exploit the distance disparity between a legitimate user and an eavesdropper, including when they lie in the same angular direction [5]. Near-field directional modulation was subsequently used to provide security in both the angular and distance domains by jointly designing analog precoding, artificial noise, and power allocation [6]. For multi-user scenarios, dynamic metasurface antennas have been employed to maximize the minimum secrecy rate in the presence of multiple eavesdroppers through joint analog and digital precoding [7]. Studies on beam focusing and power allocation have also revealed the importance of artificial noise and proposed lower-complexity algorithms for near-field XL-MIMO security [2]. Despite these advances, the secrecy gain is commonly obtained using extremely large arrays and high-dimensional beamforming technologies. Fully digital implementations require a radio-frequency (RF) chain for each active antenna, whereas hybrid or analog implementations still involve large phase-shifter networks. The resulting hardware complexity and circuit power consumption limit the practical scalability of near-field secure transceivers [6,8].

Stacked intelligent metasurfaces (SIMs) offer a power- and hardware-efficient route to alleviating this bottleneck. A SIM cascades multiple programmable metasurface layers composed of low-cost, passive elements, progressively transforming an incident electromagnetic wave and thereby performing beamforming directly in the wave domain [9]. Such wave-domain processing can reduce the reliance on conventional digital beamforming and high-resolution data converters while providing richer transformations than a single-layer metasurface [8]. These properties have recently been exploited for PLS. In particular, a SIM was designed to jointly realize modulation, beamforming, and artificial-noise generation for secure single-input single-output (SISO) transmission without introducing additional costly RF chains [10]. For multi-user multiple-input single-output (MISO) systems, the transmit beamformers and the phase shifts across the stacked layers were jointly optimized to maximize the secrecy sum rate [11]. These studies establish the security potential of SIM-based wave-domain processing, but assume a fixed feeding antenna array and hence do not exploit reconfigurable antenna geometry.

Beyond the above wave-domain enablers, RIS-type metasurfaces can also be exploited as attack surfaces rather than solely as security enablers: active RISs embed amplifiers that boost the incident signal, and this amplification enables covert pilot-spoofing attacks in which an eavesdropper corrupts the uplink channel estimation and steers downlink data toward itself, thereby undermining secrecy [12,13]. From a different perspective, RIS-aided secure communication has also been investigated at the modulation level. Because non-coherent differential chaos shift keying (DCSK) waveforms dispense with channel estimation, their interaction with a programmable RIS has been leveraged to realize secure index modulation. Specifically, a joint index-keying *M*-ary DCSK scheme was developed for RIS-aided non-coherent transmission [14], and an RIS-enabled *M*-ary DCSK scheme with block interleaving was proposed for chaotic secure communications [15]. More recently, a non-coherent RIS-empowered DCSK with two-level nested index modulation was designed to enhance spectral efficiency while preserving secrecy [16]. These studies complement the beamforming-centric secure approaches discussed above from the threat and modulation perspectives, respectively.

Movable antenna (MA) technology provides a complementary form of spatial reconfigurability by allowing antenna elements to change their positions within a confined region and thereby sample more favorable channels [17]. For secure transmission, joint optimization of the MA positions and transmit beamforming has been shown to improve the secrecy rate over fixed-position arrays [18]. MA-assisted secure MIMO designs have further combined antenna positioning, information beamforming, and artificial noise to suppress information leakage [19]. More recently, MAs have been introduced into near-field secure MIMO systems, where the range- and angle-dependent spherical-wave channel makes position optimization particularly effective even when the legitimate user and the eavesdropper are aligned in direction [20]. Nevertheless, these MA-based schemes rely primarily on active-array beamforming and do not leverage the low-power wave-domain processing capability of a SIM. Closely related to MAs, fluid antenna systems (FASs) realize spatial reconfigurability by switching a radiating element among densely packed ports within a compact region. This port-switching flexibility has been exploited for physical-layer security through analytical secrecy performance characterization [21], secret communications enabled by port selection [22], coding-enhanced cooperative jamming [23], and subsymbol precoding combined with port switching to suppress leakage toward eavesdroppers [24].

The two technologies are therefore complementary rather than substitutive: the SIM supplies dense, multilayer wave-domain degrees of freedom for shaping the radiated field, while the MAs reconfigure the array geometry and thus the channels that illuminate the SIM. Their joint design can coordinate geometric and electromagnetic-domain controls to focus energy on legitimate users and suppress leakage toward multiple eavesdroppers without relying solely on a larger number of active RF chains. However, the integration of MAs and SIMs for near-field PLS remains largely unexplored. To fill this gap, this paper proposes a secure near-field transmission scheme aided by independently movable antennas and a SIM. Under a spherical-wave channel model, the antenna positions, transmit beamformers, and SIM phase shifts are jointly optimized to maximize the secrecy sum rate in the presence of multiple eavesdroppers. Table 1 compares the related works with the proposed design from different aspects.

The main contributions of this paper are summarized as follows.

- We propose a secure near-field downlink architecture in which the base station employs *M* independently movable antennas and an *L*-layer SIM. Each antenna independently selects a continuous position along a one-dimensional rail subject to a minimum inter-antenna spacing, so that every column of the near-field MA–SIM channel is governed by a distinct position variable.
- We develop an alternating optimization framework and analyze its convergence properties. We incorporate fixed parameters into equivalent channels so that standard weighted minimum mean-square error (WMMSE) methods can be applied. The transmit beamformers are optimized using a WMMSE method with one auxiliary variable per eavesdropper stream pair, yielding a convex quadratically constrained quadratic program. The SIM phase shifts are refined layer-by-layer, using WMMSE and unit-modulus projection. The MA positions are optimized by successive convex approximation (SCA).
- We evaluate the proposed joint design through numerical simulations and quantify the individual and combined secrecy gains of independent antenna movement and the SIM under a multi-eavesdropper setting.

The remainder of this paper is organized as follows: Section 2 describes the system model and formulates the secrecy sum-rate maximization problem. Section 3 presents the joint alternating optimization framework. Numerical results are presented in Section 4.

## 2. System Model and Problem Formulation

### 2.1. Network Architecture

**Figure 1:**
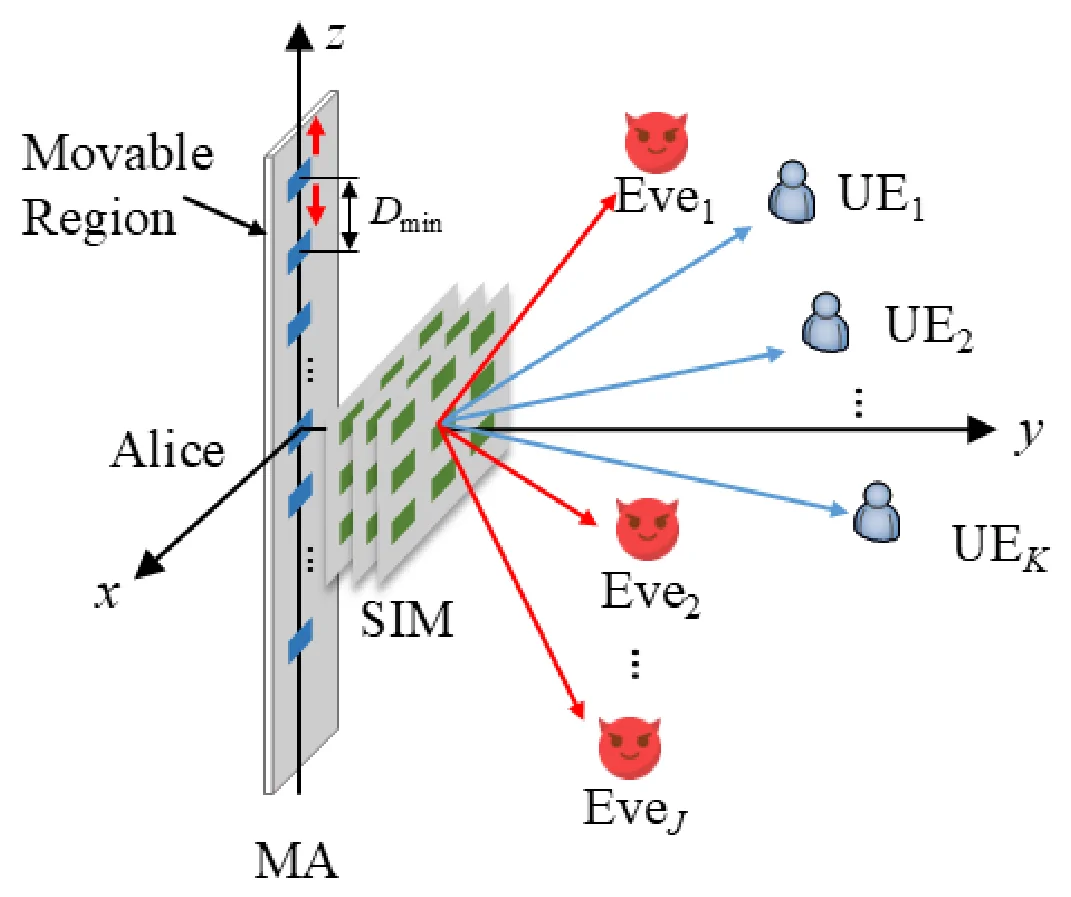
Near-field secure downlink assisted by independently movable antennas and a stacked intelligent metasurface (Red arrows: eavesdropping links. Blue arrows: legitimate links).

As shown in Figure 1, a base station (Alice) serves *K* single-antenna users over a near-field downlink, while *J* single-antenna eavesdroppers (Eves) attempt to intercept the confidential messages. Alice employs *M* independently movable antennas (MAs) and an *L*-layer stacked intelligent metasurface (SIM), with *N* transmissive elements per layer. Signals propagate from the MAs through the SIM before reaching the users and Eves. The MA positions and SIM phase shifts provide complementary spatial- and wave-domain controls for shaping the end-to-end channels and limiting information leakage. This work assumes idealized hardware. In practice, finite phase resolution, control latency, and hardware impairments of the SIM may reduce the achievable secrecy rate. In addition, MA positioning errors may arise in practice. Existing studies have reported that practical MA systems can achieve positioning precision on the order of one-hundredth or even one-thousandth of the signal wavelength [25,26]. Accordingly, we adopt an ideal continuous-position MA model, as commonly used in [17,18,19,20].

Let $q_{k}\in R^{3}$ and $q_{e,j}\in R^{3}$ denote the coordinates of user *k* and Eve *j*, respectively, where j∈J≜{1,…,J}. The position of element *n* on SIM layer *l* is denoted by $p_{l,n}\in R^{3}$. The carrier wavelength is λ.

### 2.2. MA and SIM Models

The MAs move independently along a one-dimensional rail parallel to the *z*-axis. The position of antenna *m* is

(1) $$t_{m}\left(z_{m}\right)={\left[\begin{array}{ccc} 0 & 0 & z_{m} \end{array}\right]}^{T},\;m\in M≜\left\{1,\ldots ,M\right\},$$

where $z_{m}$ is the independently adjustable position of antenna *m*. Collecting these positions gives

(2) $$z≜{\left[\begin{array}{ccc} z_{1} & \cdots & z_{M} \end{array}\right]}^{T}.$$

Without loss of generality, the antennas are indexed in ascending order along the rail, such that $-D\leq z_{1}\leq z_{2}\leq \cdots \leq z_{M}\leq D$. A minimum spacing $D_{\min}$ prevents physical overlap and limits mutual coupling: $z_{m+1}-z_{m}\geq D_{\min}$ for m=1,…,M−1.

Let $F_{1}\left(z\right)\in C^{N\times M}$ denote the channel from the MAs to the first SIM layer. The distance between antenna *m* and element *n* on the first layer is

(3) $$d_{n,m}^{\left(1\right)}\left(z_{m}\right)={∥p_{1,n}-t_{m}\left(z_{m}\right)∥}_{2}.$$

Let $\chi_{n,m}^{\left(1\right)}\left(z_{m}\right)$ be the angle between the corresponding propagation direction and the normal of the first SIM layer. Using a Rayleigh–Sommerfeld near-field propagation model [9], the (n,m)th channel coefficient is

(4) $${\left[F_{1}\left(z\right)\right]}_{n,m}=\eta_{1}\frac{A_{t}\cos\;\left(\chi_{n,m}^{\left(1\right)}\left(z_{m}\right)\right)}{d_{n,m}^{\left(1\right)}\left(z_{m}\right)}\left(\frac{1}{2\pi d_{n,m}^{\left(1\right)}\left(z_{m}\right)}+\frac{j}{\lambda }\right)\exp\;\left(-\frac{j2\pi d_{n,m}^{\left(1\right)}\left(z_{m}\right)}{\lambda }\right),$$

where $\eta_{1}\in \left(0,1\right]$ denotes the insertion-loss coefficient. Independent motion is explicit because column *m* of $F_{1}\left(z\right)$ depends only on $z_{m}$. Thus,

(5) $$F_{1}\left(z\right)=\left[\begin{array}{c} f_{1,1}\left(z_{1}\right),\cdots ,f_{1,M}\left(z_{M}\right) \end{array}\right].$$

For l=2,…,L, let $F_{l}\in C^{N\times N}$ denote the propagation matrix from layer l−1 to layer *l*. With

(6) $$d_{n,q}^{\left(l\right)}={∥p_{l,n}-p_{l-1,q}∥}_{2}\;,$$

its (n,q)th coefficient is

(7) $$\begin{array}{cc} {\left[F_{l}\right]}_{n,q}= & \eta_{l}\frac{A_{t}\cos\;\left(\chi_{n,q}^{\left(l\right)}\right)}{d_{n,q}^{\left(l\right)}}\left(\frac{1}{2\pi d_{n,q}^{\left(l\right)}}+\frac{j}{\lambda }\right)\exp\;\left(-\frac{j2\pi d_{n,q}^{\left(l\right)}}{\lambda }\right), \end{array}$$

where $\chi_{n,q}^{\left(l\right)}$ is the propagation angle relative to the normal of layer *l*. The coefficient $\eta_{l}\in \left(0,1\right]$ accounts for the insertion loss.

The phase-shift matrix of layer *l* is

(8) $$\Theta_{l}=\mathrm{diag}\;\left(\theta_{l}\right),\;\theta_{l}={\left[\begin{array}{ccc} e^{j\phi_{l,1}} & \cdots & e^{j\phi_{l,N}} \end{array}\right]}^{T},$$

where $\phi_{l,n}\in \left[0,2\pi \right)$. The equivalent transformation from the first SIM layer to the output of the last layer is

(9) $$G\left(\Theta \right)=\Theta_{L}F_{L}\cdots \Theta_{2}F_{2}\Theta_{1},$$

where $\Theta ≜{\left\{\Theta_{l}\right\}}_{l=1}^{L}$. The complete mapping from the MAs to the last SIM layer is

(10) $$B\left(z,\Theta \right)=G\left(\Theta \right)F_{1}\left(z\right)\in C^{N\times M}.$$

### 2.3. Channel and Signal Models

All legitimate users and Eves are located in the near-field region of the last SIM layer. Let $p_{L,c}$ denote the center of the last SIM layer. Define the receiver index set

(11) R≜{1,…,K}∪{(e,1),…,(e,J)}.

For r=k, let $q_{r}=q_{k}$. For r=(e,j), let $q_{r}=q_{e,j}$. The corresponding distances are

(12) $$r_{r,n}={∥q_{r}-p_{L,n}∥}_{2},\;r_{r}={∥q_{r}-p_{L,c}∥}_{2}.$$

We adopt the uniform spherical-wave (USW) model, in which $r_{r,n}$ determines the element-dependent phase while $r_{r}$ determines the common path loss. The channel from the last SIM layer to receiver *r* is

(13) $$h_{r}={\left[\begin{array}{ccc} \frac{\lambda }{4\pi r_{r}}e^{-j2\pi r_{r,1}/\lambda }\; & \ldots & \frac{\lambda }{4\pi r_{r}}e^{-j2\pi r_{r,N}/\lambda } \end{array}\right]}^{T}.$$

In particular, the channel vectors of Eve *j* and user *k* are denoted by $h_{e,j}$ and $h_{k}$, respectively. Alice transmits

(14) $$x=\sum_{i=1}^{K}v_{i}s_{i},$$

where $v_{i}\in C^{M}$ is the beamforming vector for user *i*, $s_{i}$ is the confidential symbol, and $E\{|s_{i}{|}^{2}\}=1$. The received signals at user *k* and Eve *j* are

(15) $$\begin{array}{c} y_{k}=h_{k}^{H}B\left(z,\Theta \right)\sum_{i=1}^{K}v_{i}s_{i}+n_{k}, \end{array}$$

(16) $$\begin{array}{c} y_{e,j}=h_{e,j}^{H}B\left(z,\Theta \right)\sum_{i=1}^{K}v_{i}s_{i}+n_{e,j}, \end{array}$$

for j∈J, where $n_{k}∼\mathrm{CN}\left(0,\sigma_{k}^{2}\right)$ and $n_{e,j}∼\mathrm{CN}\left(0,\sigma_{e,j}^{2}\right)$. For notational brevity, $n_{e}$ and $\sigma_{e}^{2}$ are used when all Eves are assigned the same receiver-noise variance.

The signal-to-interference-plus-noise ratio (SINR) of user *k* is

(17) $$\gamma_{k}=\frac{{\left|h_{k}^{H}B\left(z,\Theta \right)v_{k}\right|}^{2}}{\sum_{i\neq k}{\left|h_{k}^{H}B\left(z,\Theta \right)v_{i}\right|}^{2}+\sigma_{k}^{2}}.$$

We adopt a conservative non-colluding eavesdropping model in which each Eve decodes and cancels the other users’ signals before detecting $s_{k}$. The SINR of Eve *j* for message *k* is therefore

(18) $$\gamma_{e,j,k}=\frac{{\left|h_{e,j}^{H}B\left(z,\Theta \right)v_{k}\right|}^{2}}{\sigma_{e,j}^{2}},\;j\in J.$$

The leakage rate of message *k* is determined by the strongest Eve,

(19) $$\gamma_{e,k}^{\max}≜\max_{j\in J}\gamma_{e,j,k},\;R_{e,k}=\max_{j\in J}R_{e,j,k}=\log_{2}\;\left(1+\gamma_{e,k}^{\max}\right),$$

where $R_{e,j,k}=\log_{2}\left(1+\gamma_{e,j,k}\right)$. The legitimate-user rates are

(20) $$R_{k}=\log_{2}\left(1+\gamma_{k}\right),$$

and the secrecy rate of user *k* is

(21) $$R_{s,k}={\left[R_{k}-R_{e,k}\right]}^{+}.$$

The secrecy sum rate is therefore given by

(22) $$R_{\mathrm{sum}}≜\sum_{k=1}^{K}R_{s,k}.$$

### 2.4. Secrecy Sum Rate Maximization Problem

Let $V≜[v_{1},\ldots ,v_{K}]$. The transmit beamforming vectors, individual MA positions, and SIM phase shifts are jointly designed by solving

(23) $$\begin{array}{cc} \underset{V,z,\Theta }{\mathrm{maximize}}\; & R_{\mathrm{sum}}\\ \mathrm{subject}\mathrm{to}\; & C1:\sum_{k=1}^{K}{∥v_{k}∥}_{2}^{2}\leq P_{\max},\\ \; & C2:\;|{\left[\theta_{l}\right]}_{n}|=1,\;l=1,\ldots ,L,\;n=1,\ldots ,N,\\ \; & C3:-D\leq z_{1}\leq z_{2}\leq \cdots \leq z_{M}\leq D,\\ \; & C4:z_{m+1}-z_{m}\geq D_{\min},\;m=1,\ldots ,M-1. \end{array}$$

Problem (23) is nonconvex because the MA positions, transmit beamformers, and SIM phase shifts jointly determine every rate term. The unit-modulus constraints add a nonconvex feasible set, while the spacing constraints couple the MA positions. Moreover, an exhaustive search over the *M*-dimensional position vector incurs exponential growth with the search resolution. These features motivate the alternating optimization method developed next.

## 3. Joint Alternating Optimization

### 3.1. Transmit Beamforming Optimization

With the MA position vector z and SIM phase shifts Θ fixed, the transmit beamforming subproblem is

(24) $$\begin{array}{cc} \max_{V}\;\; & R_{\mathrm{sum}}\\ s.t.\;\; & \sum_{k=1}^{K}{∥v_{k}∥}_{2}^{2}\leq P_{\max}. \end{array}$$

The legitimate-user rates contain multi-user interference, whereas the leakage of each stream is determined by the strongest of the *J* Eves. We use a weighted minimum mean-square error (WMMSE) representation [27] for the legitimate-user rates and a negative-log variational representation [28] for the leakage rates. One auxiliary variable is assigned to every Eve–stream pair, so the update retains all eavesdropping constraints simultaneously.

For user *k* and Eve *j*, define the effective channels after propagation through the SIM as

(25) $${\tilde{h}}_{k}≜B{\left(z,\Theta \right)}^{H}h_{k},\;{\tilde{h}}_{e,j}≜B{\left(z,\Theta \right)}^{H}h_{e,j},$$

where both vectors belong to $C^{M}$. The end-to-end scalar channels for stream *i* are ${\tilde{h}}_{k}^{H}v_{i}$ and ${\tilde{h}}_{e,j}^{H}v_{i}$. Let user *k* estimate $s_{k}$ as ${\hat{s}}_{k}=u_{k}^{*}y_{k}$, where $u_{k}\in C$ is a scalar receive filter. The corresponding mean-square error (MSE) is

(26) $$e_{k}={\left|u_{k}\right|}^{2}\left(\sum_{i=1}^{K}{\left|{\tilde{h}}_{k}^{H}v_{i}\right|}^{2}+\sigma_{k}^{2}\right)-2ℜ\;\left\{u_{k}^{*}{\tilde{h}}_{k}^{H}v_{k}\right\}+1.$$

For a fixed beamforming matrix, the minimum-MSE receiver and the resulting MSE are

(27) $$\begin{array}{c} u_{k}^{⋆}=\frac{{\tilde{h}}_{k}^{H}v_{k}}{\sum_{i=1}^{K}{\left|{\tilde{h}}_{k}^{H}v_{i}\right|}^{2}+\sigma_{k}^{2}}, \end{array}$$

(28) $$\begin{array}{c} e_{k}^{⋆}=1-\frac{|{\tilde{h}}_{k}^{H}v_{k}{|}^{2}}{\sum_{i=1}^{K}{\left|{\tilde{h}}_{k}^{H}v_{i}\right|}^{2}+\sigma_{k}^{2}}=\frac{1}{1+\gamma_{k}}. \end{array}$$

Introducing a positive WMMSE weight $w_{k}$ gives the exact identity [27]

(29) $$R_{k}=\max_{u_{k}\in C,\;w_{k}>0}\;\frac{1}{\ln2}\left(\ln w_{k}-w_{k}e_{k}+1\right).$$

The maximizing receiver is given by (27), and the corresponding weight is

(30) $$w_{k}^{⋆}={\left(e_{k}^{⋆}\right)}^{-1}.$$

For each Eve–stream pair (j,k), the negative leakage rate admits a complementary variational representation. For any x>0, the identity

(31) $$-\ln x=\max_{a>0}\left(\ln a-ax+1\right)$$

holds with $a^{⋆}=x^{-1}$ [28]. Applying it to $x=1+\gamma_{e,j,k}$ yields

(32) $$\begin{array}{c} -R_{e,j,k}=\max_{a_{j,k}>0}\;\frac{1}{\ln2}\left[\ln a_{j,k}-a_{j,k}\left(1+\frac{|{\tilde{h}}_{e,j}^{H}v_{k}{|}^{2}}{\sigma_{e,j}^{2}}\right)+1\right], \end{array}$$

with

(33) $$a_{j,k}^{⋆}={\left(1+\frac{|{\tilde{h}}_{e,j}^{H}v_{k}{|}^{2}}{\sigma_{e,j}^{2}}\right)}^{-1}.$$

For fixed $a_{j,k}>0$, define the concave quadratic function of $v_{k}$

(34) $$\begin{array}{c} \ell_{j,k}\left(v_{k},a_{j,k}\right)≜\frac{1}{\ln2}\left[\ln a_{j,k}-a_{j,k}+1-\frac{a_{j,k}}{\sigma_{e,j}^{2}}{\left|{\tilde{h}}_{e,j}^{H}v_{k}\right|}^{2}\right], \end{array}$$

which satisfies $\ell_{j,k}\leq -R_{e,j,k}$ for every $a_{j,k}>0$, with equality at (33). Since the strongest Eve determines the leakage rate,

(35) $$-R_{e,k}=-\max_{j\in J}R_{e,j,k}=\min_{j\in J}\left\{-R_{e,j,k}\right\}.$$

Therefore, $\min_{j\in J}\ell_{j,k}$ is a concave lower bound on $-R_{e,k}$ for fixed ${\left\{a_{j,k}\right\}}_{j=1}^{J}$ and is tight at the current beamforming matrix when all $a_{j,k}$ are updated using (33).

Combining this worst-Eve lower bound with the WMMSE representation gives

(36) $$\begin{array}{c} {\underline{R}}_{\mathrm{sum}}\left(V,u,w,A\right)≜\sum_{k=1}^{K}\left[\frac{\ln w_{k}-w_{k}e_{k}+1}{\ln2}+\min_{j\in J}\ell_{j,k}\left(v_{k},a_{j,k}\right)\right], \end{array}$$

where $u≜{\left[u_{1},\ldots ,u_{K}\right]}^{T}$, $w≜{\left[w_{1},\ldots ,w_{K}\right]}^{T}$, and $A≜\left[a_{j,k}\right]\in R_{++}^{J\times K}$. For every feasible set of auxiliary variables,

(37) $${\underline{R}}_{\mathrm{sum}}\left(V,u,w,A\right)\leq R_{\mathrm{sum}}\left(V\right),$$

and equality holds at the current V after updating u, w, and A according to (27), (30), and (33), respectively. This tight lower-bound construction will also be used for the SIM phase-shift update.

Fix the auxiliary variables at their current optimal values and introduce $\xi_{k}\in R$ to represent the scaled worst-Eve lower bound for stream *k*. After multiplying the objective by ln2 and removing terms independent of V and ξ, define

(38) $$\begin{array}{c} Q≜\sum_{i=1}^{K}w_{i}{\left|u_{i}\right|}^{2}{\tilde{h}}_{i}{\tilde{h}}_{i}^{H}, \end{array}$$

(39) $$\begin{array}{c} \alpha_{k}≜w_{k}u_{k}{\tilde{h}}_{k}, \end{array}$$

(40) $$\begin{array}{c} c_{j,k}≜\ln a_{j,k}-a_{j,k}+1. \end{array}$$

Because Q⪰0, the beamforming update is the following convex quadratically constrained quadratic program (QCQP):

(41) $$\begin{array}{cc} \underset{V,\xi }{\mathrm{maximize}} & \sum_{k=1}^{K}\left[2ℜ\;\left\{\alpha_{k}^{H}v_{k}\right\}-v_{k}^{H}Qv_{k}+\xi_{k}\right]\\ s.t. & C1:\sum_{k=1}^{K}{∥v_{k}∥}_{2}^{2}\leq P_{\max},\\ \; & C2:\xi_{k}\leq c_{j,k}-\frac{a_{j,k}}{\sigma_{e,j}^{2}}{\left|{\tilde{h}}_{e,j}^{H}v_{k}\right|}^{2},\;j=1,\ldots ,J,\;k=1,\ldots ,K. \end{array}$$

Constraint C2 in(41) is the hypograph of a concave quadratic function. Hence, the feasible set of (41) is convex, and the problem can be solved directly using CVX.

For fixed $V^{\left(t\right)}$, the auxiliary-variable updates make (37) tight. Solving (41) then maximizes this tight concave lower bound, so

(42) $$R_{\mathrm{sum}}\left(V^{\left(t+1\right)}\right)\geq {\underline{R}}_{\mathrm{sum}}\left(V^{\left(t+1\right)}\right)\geq {\underline{R}}_{\mathrm{sum}}\left(V^{\left(t\right)}\right)=R_{\mathrm{sum}}\left(V^{\left(t\right)}\right).$$

Thus, the true worst-Eve secrecy sum rate is nondecreasing. The transmit-power constraint makes it bounded above, and the objective sequence therefore converges.

### 3.2. SIM Phase-Shift Optimization

With the transmit beamforming matrix V and MA position vector z fixed, the SIM phase-shift subproblem is

(43) $$\begin{array}{cc} \max_{\Theta } & R_{\mathrm{sum}}\\ s.t. & |{\left[\theta_{l}\right]}_{n}|=1,\;l=1,\ldots ,L,\;n=1,\ldots ,N. \end{array}$$

The phase-shift matrices remain coupled through the cascaded transformation G(Θ). We address this coupling by updating one SIM layer at a time.

When layer *l* is updated, all other phase-shift matrices remain fixed. We separate the output-side and input-side cascades as

(44) $$\begin{array}{cc} G_{l}^{\mathrm{out}} & ≜\left\{\begin{array}{cc} I_{N}, & l=L,\\ \Theta_{L}F_{L}\cdots \Theta_{l+1}F_{l+1}, & l<L, \end{array}\right. \end{array}$$

(45) $$\begin{array}{cc} G_{l}^{\mathrm{in}}\left(z\right) & ≜\left\{\begin{array}{cc} F_{1}\left(z\right), & l=1,\\ F_{l}\Theta_{l-1}F_{l-1}\cdots \Theta_{1}F_{1}\left(z\right), & l>1. \end{array}\right. \end{array}$$

Here, $G_{l}^{\mathrm{out}}\in C^{N\times N}$ and $G_{l}^{\mathrm{in}}\left(z\right)\in C^{N\times M}$. This decomposition expresses the end-to-end mapping in (10) as

(46) $$B\left(z,\Theta \right)=G_{l}^{\mathrm{out}}\Theta_{l}G_{l}^{\mathrm{in}}\left(z\right).$$

For user *k*, Eve *j*, and data stream *i*, define the phase-domain effective channel vectors $\psi_{k,i}^{\left(l\right)}\in C^{N}$ and $\psi_{e,j,i}^{\left(l\right)}\in C^{N}$ as

(47) $$\begin{array}{c} \psi_{k,i}^{\left(l\right)}≜\left({\left(G_{l}^{\mathrm{out}}\right)}^{H}h_{k}\right)⊙{\left(G_{l}^{\mathrm{in}}\left(z\right)v_{i}\right)}^{*}, \end{array}$$

(48) $$\begin{array}{c} \psi_{e,j,i}^{\left(l\right)}≜\left({\left(G_{l}^{\mathrm{out}}\right)}^{H}h_{e,j}\right)⊙{\left(G_{l}^{\mathrm{in}}\left(z\right)v_{i}\right)}^{*}. \end{array}$$

Because $\Theta_{l}=\mathrm{diag}\left(\theta_{l}\right)$, both effective scalar channels are linear in the layer-*l* phase vector:

(49) $$h_{k}^{H}B\left(z,\Theta \right)v_{i}={\left(\psi_{k,i}^{\left(l\right)}\right)}^{H}\theta_{l},\;h_{e,j}^{H}B\left(z,\Theta \right)v_{i}={\left(\psi_{e,j,i}^{\left(l\right)}\right)}^{H}\theta_{l}.$$

For the current $\theta_{l}$, we evaluate $u_{k}$, $e_{k}$, $w_{k}$, and every $a_{j,k}$ from (27), (28), (30), and (33), respectively. In these expressions, the end-to-end scalar channels are replaced by their layer-*l* forms in (49). This substitution applies the common multiple-Eve lower bound in (36) without repeating the WMMSE derivation.

Fixing the auxiliary variables at their current optima makes that lower bound tight at the current phase vector. After multiplying it by ln2 and omitting terms independent of $\theta_{l}$, define

(50) $$\begin{array}{c} O_{u,l}≜\sum_{k=1}^{K}w_{k}{\left|u_{k}\right|}^{2}\sum_{i=1}^{K}\psi_{k,i}^{\left(l\right)}{\left(\psi_{k,i}^{\left(l\right)}\right)}^{H}, \end{array}$$

(51) $$\begin{array}{c} b_{l}≜\sum_{k=1}^{K}w_{k}u_{k}\psi_{k,k}^{\left(l\right)}, \end{array}$$

(52) $$\begin{array}{c} Z_{j,k}^{\left(l\right)}≜\frac{a_{j,k}}{\sigma_{e,j}^{2}}\psi_{e,j,k}^{\left(l\right)}{\left(\psi_{e,j,k}^{\left(l\right)}\right)}^{H}. \end{array}$$

All matrices $O_{u,l}$ and $Z_{j,k}^{\left(l\right)}$ are Hermitian positive semidefinite. Using $c_{j,k}$ from (40) and introducing a real slack variable $\xi_{k}$ for each stream gives the layer-*l* lower-bound problem

(53) $$\begin{array}{cc} \underset{\theta_{l},\xi }{\mathrm{maximize}} & 2ℜ\;\left\{\theta_{l}^{H}b_{l}\right\}-\theta_{l}^{H}O_{u,l}\theta_{l}+\sum_{k=1}^{K}\xi_{k}\\ \mathrm{subject}\mathrm{to} & C1:\xi_{k}\leq c_{j,k}-\theta_{l}^{H}Z_{j,k}^{\left(l\right)}\theta_{l},\;j=1,\ldots ,J,\;k=1,\ldots ,K,\\ \; & C2:\;|{\left[\theta_{l}\right]}_{n}|=1,\;n=1,\ldots ,N. \end{array}$$

The objective is concave and C1 in (53) defines a convex hypograph, but the unit-modulus constraint remains nonconvex. We first solve the convex relaxation

(54) $$|{\left[{\tilde{\theta }}_{l}\right]}_{n}|\leq 1,\;n=1,\ldots ,N,$$

where ${\tilde{\theta }}_{l}$ replaces $\theta_{l}$ in (53). This relaxed QCQP can be solved directly by CVX.

At layer-update iteration *t*, let ${\tilde{\theta }}_{l}^{⋆}$ be the relaxed solution. Its element-wise unit-modulus projection is

(55) $${\left[{\hat{\theta }}_{l}\right]}_{n}=\left\{\begin{array}{cc} \exp\;\left(j\arg({\left[{\tilde{\theta }}_{l}^{⋆}\right]}_{n})\right),\; & |{\left[{\tilde{\theta }}_{l}^{⋆}\right]}_{n}|>0,\\ {}[\theta_{l}^{\left(t\right)}{]}_{n},\; & |{\left[{\tilde{\theta }}_{l}^{⋆}\right]}_{n}|=0. \end{array}\right.$$

Because projection can reduce the objective, the projected vector is not accepted automatically. We instead use the unit-modulus interpolation

(56) $$\theta_{l}\left(\tau \right)=\exp\;\left(j\arg\;\left(\left(1-\tau \right)\theta_{l}^{\left(t\right)}+\tau {\hat{\theta }}_{l}\right)\right),\;\tau \in \left(0,1\right],$$

where the exponential and argument are applied element-wise. Starting from τ=1, we reduce τ geometrically until the true multiple-Eve secrecy sum rate does not decrease. If no positive trial step is accepted, the current phase vector is retained. This safeguard preserves the original relaxed-CVX and projection structure while making the layer update monotonic for the worst-Eve objective.

The auxiliary-variable update makes the multiple-Eve lower bound tight at the current phase vector. The relaxed QCQP optimizes this bound over a superset of the unit-modulus feasible set. The projected line search, however, accepts a phase vector only when the true worst-Eve secrecy sum rate does not decrease. The resulting layer-wise objective sequence is therefore monotonic and bounded above, and hence convergent.

### 3.3. Joint Optimization of the Movable-Antenna Positions

With the transmit beamforming matrix V and the SIM phase shifts Θ fixed, the MA position subproblem is

(57) $$\max_{z\in Z}\;R_{\mathrm{sum}}\left(z\right),$$

where the feasible position set is

(58) $$Z≜\left\{z\in R^{M}:-D\leq z_{1},\;z_{m+1}-z_{m}\geq D_{\min},\;z_{M}\leq D\right\}.$$

The set Z is a closed and convex polytope. It is nonempty when $\left(M-1\right)D_{\min}\leq 2D$. Although each position controls one column of $F_{1}\left(z\right)$, the entries of z remain coupled through multi-user interference and the spacing constraints. We therefore update all MA positions jointly.

For a legitimate receiver r=k or an eavesdropper r=(e,j) and stream *i*, define the position-dependent scalar channel

(59) $$\zeta_{r,i}\left(z\right)≜h_{r}^{H}G\left(\Theta \right)F_{1}\left(z\right)v_{i},$$

where $h_{r}=h_{k}$ for r=k and $h_{r}=h_{e,j}$ for r=(e,j). The column structure in (5) yields

(60) $$\frac{\partial \zeta_{r,i}}{\partial z_{m}}={\left[v_{i}\right]}_{m}h_{r}^{H}G\left(\Theta \right)f_{1,m}^{\prime }\left(z_{m}\right),$$

where $f_{1,m}^{\prime }\left(z_{m}\right)≜\partial f_{1,m}\left(z_{m}\right)/\partial z_{m}$.

For completeness, this derivative can be obtained directly from the Rayleigh–Sommerfeld coefficient. Let $n_{1}$ be the unit normal of the first SIM layer, let $e_{z}={\left[0,0,1\right]}^{T}$, and define

(61) $$\kappa_{n,m}\left(z_{m}\right)≜\cos\;\left(\chi_{n,m}^{\left(1\right)}\left(z_{m}\right)\right),\;k_{0}≜\frac{2\pi }{\lambda }.$$

The derivatives of the propagation distance and directional factor are

(62) $$\begin{array}{c} {\left(d_{n,m}^{\left(1\right)}\right)}^{\prime }\left(z_{m}\right)=\frac{z_{m}-{\left[p_{1,n}\right]}_{3}}{d_{n,m}^{\left(1\right)}\left(z_{m}\right)}, \end{array}$$

(63) $$\begin{array}{c} \kappa_{n,m}^{\prime }\left(z_{m}\right)=-\frac{n_{1}^{T}e_{z}}{d_{n,m}^{\left(1\right)}\left(z_{m}\right)}-\frac{\kappa_{n,m}\left(z_{m}\right){\left(d_{n,m}^{\left(1\right)}\right)}^{\prime }\left(z_{m}\right)}{d_{n,m}^{\left(1\right)}\left(z_{m}\right)}=-\frac{\kappa_{n,m}\left(z_{m}\right){\left(d_{n,m}^{\left(1\right)}\right)}^{\prime }\left(z_{m}\right)}{d_{n,m}^{\left(1\right)}\left(z_{m}\right)}. \end{array}$$

Define the non-exponential factor

(64) $$\beta_{n,m}\left(z_{m}\right)≜\eta_{1}A_{t}\kappa_{n,m}\left(z_{m}\right)\left(\frac{1}{2\pi {\left[d_{n,m}^{\left(1\right)}\left(z_{m}\right)\right]}^{2}}+\frac{j}{\lambda d_{n,m}^{\left(1\right)}\left(z_{m}\right)}\right).$$

Then ${\left[f_{1,m}\left(z_{m}\right)\right]}_{n}=\beta_{n,m}\left(z_{m}\right)e^{-jk_{0}d_{n,m}^{\left(1\right)}\left(z_{m}\right)}$, and

(65) $${\left[f_{1,m}^{\prime }\left(z_{m}\right)\right]}_{n}=e^{-jk_{0}d_{n,m}^{\left(1\right)}\left(z_{m}\right)}\left[\beta_{n,m}^{\prime }\left(z_{m}\right)-jk_{0}\beta_{n,m}\left(z_{m}\right){\left(d_{n,m}^{\left(1\right)}\right)}^{\prime }\left(z_{m}\right)\right],$$

where

(66) $$\begin{array}{cc} \beta_{n,m}^{\prime }\left(z_{m}\right)= & \eta_{1}A_{t}\kappa_{n,m}^{\prime }\left(z_{m}\right)\left(\frac{1}{2\pi {\left[d_{n,m}^{\left(1\right)}\right]}^{2}}+\frac{j}{\lambda d_{n,m}^{\left(1\right)}}\right)\\ \; & +\eta_{1}A_{t}\kappa_{n,m}\left(z_{m}\right)\left(-\frac{1}{\pi {\left[d_{n,m}^{\left(1\right)}\right]}^{3}}-\frac{j}{\lambda {\left[d_{n,m}^{\left(1\right)}\right]}^{2}}\right){\left(d_{n,m}^{\left(1\right)}\right)}^{\prime }\left(z_{m}\right). \end{array}$$

For compactness, every distance without an explicit argument in (66) is evaluated at $z_{m}$.

Let $\omega_{r,i}\left(z\right)≜{\left|\zeta_{r,i}\left(z\right)\right|}^{2}$. Its position derivative is

(67) $$\frac{\partial \omega_{r,i}}{\partial z_{m}}=2ℜ\;\left\{\zeta_{r,i}^{*}\frac{\partial \zeta_{r,i}}{\partial z_{m}}\right\}.$$

For user *k*, define

(68) $$T_{k}≜\sum_{i=1}^{K}\omega_{k,i}+\sigma_{k}^{2},\;I_{k}≜\sum_{i\neq k}\omega_{k,i}+\sigma_{k}^{2}.$$

For Eve *j* and stream *k*, additionally define

(69) $$E_{j,k}≜\sigma_{e,j}^{2}+\omega_{e,j,k}.$$

The gradients of the legitimate-user rate and each individual leakage rate are

(70) $$\begin{array}{cc} \; & \nabla_{z}R_{k}=\frac{1}{\ln2}\left(\frac{\nabla_{z}T_{k}}{T_{k}}-\frac{\nabla_{z}I_{k}}{I_{k}}\right), \end{array}$$

(71) $$\begin{array}{cc} \; & \nabla_{z}R_{e,j,k}=\frac{1}{\ln2}\frac{\nabla_{z}\omega_{e,j,k}}{E_{j,k}}. \end{array}$$

Here, $\omega_{e,j,k}={\left|\zeta_{e,j,k}\right|}^{2}$, and all power gradients follow from (67). The worst-Eve function $R_{e,k}=\max_{j\in J}R_{e,j,k}$ is generally nondifferentiable when the identity of the strongest Eve changes. Directly substituting the gradient of one preselected Eve into a conventional gradient update can therefore fail at such switching points.

To handle the maximum exactly, introduce a real variable $\nu_{k}$ for each stream and rewrite (57) equivalently as

(72) $$\begin{array}{cc} \underset{z,\nu }{\mathrm{maximize}} & \sum_{k=1}^{K}\left(R_{k}\left(z\right)+\nu_{k}\right)\\ \mathrm{subject}\mathrm{to} & C1:z\in Z,\\ \; & C2:\nu_{k}\leq -R_{e,j,k}\left(z\right),\;j=1,\ldots ,J,\;k=1,\ldots ,K. \end{array}$$

At the optimum, $\nu_{k}=-\max_{j}R_{e,j,k}$, so all Eve constraints are retained rather than replacing them by a sum or fixing one Eve in advance.

For fixed beamformers and SIM phase shifts, let f(z) denote any legitimate-user rate $R_{k}\left(z\right)$ or individual eavesdropping rate $R_{e,j,k}\left(z\right)$. Assume that Z is nonempty. The rail bounds and non-strict linear spacing constraints make it closed, bounded, and convex, and hence compact. Under the adopted channel model, propagation distances bounded away from zero and positive receiver noise powers ensure that *f* is twice continuously differentiable on a neighborhood of Z. Thus, $L_{f}=\max_{z\in Z}{∥\nabla_{z}^{2}f\left(z\right)∥}_{2}<\infty$. Integrating the Hessian along the segment between any x,y∈Z gives ${∥\nabla f\left(y\right)-\nabla f\left(x\right)∥}_{2}\leq L_{f}{∥y-x∥}_{2}$. This property applies to every rate component used in the SCA bounds. At a feasible expansion point $z^{\left(t\right)}$, define $d=z-z^{\left(t\right)}$ and the concave quadratic surrogate functions

(73) $$\begin{array}{c} {\hat{R}}_{k}\left(z|z^{\left(t\right)}\right)≜R_{k}\left(z^{\left(t\right)}\right)+\nabla_{z}R_{k}{\left(z^{\left(t\right)}\right)}^{T}d-\frac{\mu_{t}}{2}{∥d∥}_{2}^{2}, \end{array}$$

(74) $$\begin{array}{c} {\hat{R}}_{e,j,k}^{-}\left(z|z^{\left(t\right)}\right)≜-R_{e,j,k}\left(z^{\left(t\right)}\right)-\nabla_{z}R_{e,j,k}{\left(z^{\left(t\right)}\right)}^{T}d-\frac{\mu_{t}}{2}{∥d∥}_{2}^{2}. \end{array}$$

If $\mu_{t}$ is no smaller than the largest Lipschitz constant among these component gradients, the descent lemma gives ${\hat{R}}_{k}\leq R_{k}$ and ${\hat{R}}_{e,j,k}^{-}\leq -R_{e,j,k}$ throughout Z. For any $\mu_{t}>0$, each surrogate matches the value and gradient of its smooth rate component at $z^{\left(t\right)}$. The multiple-Eve SCA update is the convex QCQP

(75) $$\begin{array}{cc} \underset{z,\nu }{\mathrm{maximize}} & \sum_{k=1}^{K}\left({\hat{R}}_{k}\left(z|z^{\left(t\right)}\right)+\nu_{k}\right)\\ \mathrm{subject}\mathrm{to} & C1:z\in Z,\\ \; & C2:\nu_{k}\leq {\hat{R}}_{e,j,k}^{-}\left(z|z^{\left(t\right)}\right),\;j=1,\ldots ,J,\;k=1,\ldots ,K. \end{array}$$

The objective is concave, and every constraint in C2 is the hypograph of a concave quadratic function. Hence, (75) is a convex optimization problem.

The Lipschitz constants need not be known explicitly. For a fixed expansion point $z^{\left(t\right)}$, the algorithm starts from a positive trial curvature, solves (75), and verifies at the candidate $\tilde{z}$ that

(76) $$\begin{array}{ccc} R_{k}\left(\tilde{z}\right)\geq {\hat{R}}_{k}\left(\tilde{z}|z^{\left(t\right)}\right), & \; & k=1,\ldots ,K, \end{array}$$

(77) $$\begin{array}{ccc} -R_{e,j,k}\left(\tilde{z}\right)\geq {\hat{R}}_{e,j,k}^{-}\left(\tilde{z}|z^{\left(t\right)}\right),\; & \; & j=1,\ldots ,J,\;k=1,\ldots ,K. \end{array}$$

These inequalities are checked for every legitimate-user and Eve-rate component and are sufficient for feasibility and objective monotonicity at the candidate. If either test fails, $\mu_{t}$ is replaced by $\varrho \mu_{t}$ without changing $z^{\left(t\right)}$, and the QCQP is solved again. This procedure remains valid when the identity of the strongest Eve changes between iterations.

**Proposition** **1.** *Suppose that Z is nonempty, the MA–SIM distances are bounded away from zero, the gradients of all smooth component rates are Lipschitz continuous on Z, and each SCA subproblem is solved exactly. If the candidate passes (76) and (77), the proposed SCA position update is feasible and does not decrease the worst-Eve secrecy sum rate. Because the objective is bounded above on the compact feasible set, the sequence of accepted objective values converges.*

**Proof.**  At $z^{\left(t\right)}$, set $\nu_{k}^{\left(t\right)}=-\max_{j}R_{e,j,k}\left(z^{\left(t\right)}\right)$. The current point is feasible for (75) because every surrogate matches its corresponding rate component there. Optimality of $\left(z^{\left(t+1\right)},\nu^{\left(t+1\right)}\right)$ and the candidate-wise lower-bound conditions give

(78) $$\begin{array}{cc} R_{\mathrm{sum}}\left(z^{\left(t+1\right)}\right) & \geq \sum_{k=1}^{K}\left[{\hat{R}}_{k}\left(z^{\left(t+1\right)}|z^{\left(t\right)}\right)+\nu_{k}^{\left(t+1\right)}\right]\\ \; & \geq \sum_{k=1}^{K}\left[{\hat{R}}_{k}\left(z^{\left(t\right)}|z^{\left(t\right)}\right)+\nu_{k}^{\left(t\right)}\right]=R_{\mathrm{sum}}\left(z^{\left(t\right)}\right). \end{array}$$

The continuous objective is bounded above on compact Z, so the sequence of accepted objective values converges.    □

### 3.4. Overall Algorithm and Analysis

The three blocks are assembled in Algorithm 1. Each outer iteration updates the transmit beamformers, the MA positions, and then the SIM phase shifts. The true objective $R_{\mathrm{sum}}$ is used to accept every candidate update.
**Algorithm 1** Joint MA–SIM Alternating Optimization Against Multiple Eves**Require:** Feasible $V^{\left(0\right)}$, $z^{\left(0\right)}$, and $\Theta^{\left(0\right)}$

**Require:** Tolerances $\varepsilon_{v}$, $\varepsilon_{z}$, $\varepsilon_{\phi }$, and $\varepsilon_{\mathrm{AO}}$; limits $T_{v}$, $T_{z}$, $T_{\phi }^{\mathrm{in}}$, $T_{\mathrm{AO}}$, and $B_{z}$

**Require:** Initial curvature $\mu_{0}>0$ and backtracking factor ϱ>1
  1:Set q←0 and evaluate $R_{\mathrm{sum}}^{\left(0\right)}=R_{\mathrm{sum}}\left(V^{\left(0\right)},z^{\left(0\right)},\Theta^{\left(0\right)}\right)$  2:**repeat**  3:      Fix $z^{\left(q\right)}$ and $\Theta^{\left(q\right)}$; update $\left\{u_{k},w_{k},a_{j,k}\right\}$ and solve (41) until the beamforming stopping rule is met or $T_{v}$ iterations are reached  4:      If the beamforming candidate decreases the true $R_{\mathrm{sum}}$, retain $V^{\left(q\right)}$; otherwise accept it; denote the resulting point by $V^{\left(q+1\right)}$  5:      Set $z_{p}^{\left(0\right)}\leftarrow z^{\left(q\right)}$ and t←0, with $V^{\left(q+1\right)}$ and $\Theta^{\left(q\right)}$ fixed  6:      **repeat**  7:            Evaluate the rates and position gradients at $z_{p}^{\left(t\right)}$ and set $\mu \leftarrow \mu_{0}$  8:            **repeat**  9:                  Solve (75) around $z_{p}^{\left(t\right)}$ using the current μ10:                  If the candidate fails (76) or (77), set μ←ϱμ11:            **until** both lower-bound tests hold or $B_{z}$ trials are reached12:            If no candidate passes, retain $z_{p}^{\left(t\right)}$ and terminate the position update13:            If the candidate decreases the true $R_{\mathrm{sum}}$, retain $z_{p}^{\left(t\right)}$ and terminate the position update14:            Otherwise, set it as $z_{p}^{\left(t+1\right)}$ and increment *t*15:      **until** $∥z_{p}^{\left(t\right)}-z_{p}^{\left(t-1\right)}{∥}_{2}\leq \varepsilon_{z}$ or $t=T_{z}$16:      Set $z^{\left(q+1\right)}\leftarrow z_{p}^{\left(t\right)}$ and $\Theta^{\left(q+1\right)}\leftarrow \Theta^{\left(q\right)}$17:      **for** l=1,…,L **do**18:            **repeat**19:                  Form $G_{l}^{\mathrm{out}}$ and $G_{l}^{\mathrm{in}}$; update $\left\{u_{k},w_{k},a_{j,k}\right\}$ using the current $\Theta^{\left(q+1\right)}$20:                  Form the phase-domain quantities, solve the relaxed form of (53), project it using (55), and apply (56)21:                  Accept the first unit-modulus candidate that does not decrease the true $R_{\mathrm{sum}}$; otherwise retain layer *l*22:            **until** the layer-*l* stopping rule is met or $T_{\phi }^{\mathrm{in}}$ updates are reached23:      **end for**24:      Evaluate $R_{\mathrm{sum}}^{\left(q+1\right)}=R_{\mathrm{sum}}\left(V^{\left(q+1\right)},z^{\left(q+1\right)},\Theta^{\left(q+1\right)}\right)$ and set q←q+125:**until** $|R_{\mathrm{sum}}^{\left(q\right)}-R_{\mathrm{sum}}^{\left(q-1\right)}|\leq \varepsilon_{\mathrm{AO}}$ or $q=T_{\mathrm{AO}}$
**Ensure:**
$V^{⋆}$, $z^{⋆}$, $\Theta^{⋆}$, and $R_{\mathrm{sum}}^{⋆}$


*Convergence Analysis:* The beamforming update maximizes a current-point-tight lower bound. The position candidate is accepted only after the lower-bound inequalities hold for every legitimate-user and Eve-rate component. The phase update applies an explicit acceptance test after unit-modulus projection. Consequently, every accepted block update preserves feasibility and does not decrease the true secrecy sum rate. Thus, the accepted objective-value sequence is nondecreasing and bounded above, and hence convergent. Because the feasible set is compact, the iterate sequence has at least one limit point. However, this does not imply that the limit point is stationary for the original nonconvex problem.

*Computational Complexity Analysis:* The interior-point complexity of a convex problem with *n* real variables and *m* inequality constraints is denoted by $P_{\mathrm{IP}}\left(n,m\right)=O\;\left(\sqrt{m}\;\left(m+n\right)n^{2}\right)$. The beamforming, MA-position, and per-layer SIM QCQPs have $\left(n_{v},m_{v}\right)=\left(2KM+K,JK+1\right)$, $\left(n_{z},m_{z}\right)=\left(M+K,JK+M+1\right)$, and $\left(n_{\phi },m_{\phi }\right)=\left(2N+K,JK+N\right)$, respectively. The complexity of the proposed algorithm is

(79) $$C_{\mathrm{AO}}=O\;\left\{T_{\mathrm{AO}}\left[T_{v}P_{\mathrm{IP}}\left(n_{v},m_{v}\right)+T_{z}B_{z}P_{\mathrm{IP}}\left(n_{z},m_{z}\right)+LT_{\phi }^{\mathrm{in}}P_{\mathrm{IP}}\left(n_{\phi },m_{\phi }\right)\right]\right\},$$

where $B_{z}$ accounts for repeated MA-QCQP solves during curvature backtracking. For implementations that solve the convex subproblems through CVX, efficient backend solvers that exploit problem structure could further reduce the practical computational cost.

## 4. Simulation Results

This section evaluates the performance of the proposed scheme. Unless otherwise specified, the carrier frequency is f=28 GHz. Alice employs M=7 independently movable antennas and a SIM with L=2 layers and N=80 elements per layer. Each SIM layer uses a linear array. The first SIM layer is located at $d_{1}=0.05$ m, and the layer spacing is $d_{2}=\lambda /2$. The insertion-loss coefficients are set to $\eta_{l}=0.9$ for l=1,…,L. Each MA moves along the *z*-axis within [−D,D]=[−0.08,0.08] m, subject to $D_{\min}=\lambda /2$. The fixed uniform linear array (ULA) used as a benchmark is centered at the origin with half-wavelength spacing.

The main multi-receiver experiment considers K=2 legitimate users and J=2 non-colluding Eves in the *y*–*z* plane. Their polar coordinates, written as (r,ϑ) with the angle measured from the positive *y*-axis toward the positive *z*-axis, are $q_{1}=\left(6\;m,-20^{\circ }\right)$, $q_{2}=\left(6\;m,17^{\circ }\right)$, and $q_{e,1}=\left(3\;m,-20^{\circ }\right)$, $q_{e,2}=\left(3\;m,17^{\circ }\right)$, respectively. Thus, each Eve is closer to Alice and aligned with one user, which creates a demanding range-discrimination problem. The noise powers are normalized to $\sigma_{k}^{2}=\sigma_{e,j}^{2}=1$. Accordingly, a reported power of *P* dB denotes $10\log_{10}\left(P_{\max}\right)$ in the normalized model and should not be interpreted as dBm.

The outer alternating optimization loop is capped at 50 iterations and uses a stopping tolerance of $10^{-4}$ in the experiments. Each beamforming, MA-position, and SIM-layer update also has a maximum of 50 inner iterations and uses an absolute objective change tolerance of $10^{-4}$. A feasible initial beamformer is obtained by normalizing an all-ones matrix $\tilde{V}$ as $V^{\left(0\right)}=\sqrt{P_{\max}}\tilde{V}/{∥\tilde{V}∥}_{F}$. Our simulations show that the MA position initialization affects the final secrecy sum rate, whereas the SIM phase initialization has little influence. Uniformly distributing the MAs along the rail yields relatively good performance in the tested cases. We therefore adopt this MA initialization in our simulations and initialize all SIM phase angles to zero. The simulations are conducted using MATLAB R2021b on an Intel Core i7-11700 (2.50 GHz) personal computer with 32 GB RAM. Three sequential 50-iteration runs of the CVX implementation required 564.7, 553.2, and 561.0 s, respectively, giving a mean of 559.6 s.

Four schemes are compared under the same parameter settings. These baselines are intended to compare system configurations rather than to establish algorithmic superiority.

- *Proposed MA + SIM*: jointly optimizes the transmit beamformers, all individual MA positions, and all SIM phase shifts.
- *Fixed ULA + SIM*: fixes the *M* antennas as a centered half-wavelength ULA and optimizes only the beamformers and SIM phases.
- *MA only*: removes the SIM and jointly optimizes the transmit beamformers and the *M* individual MA positions.
- *SIM only*: uses one fixed antenna at the origin with equal power allocation across the streams and optimizes only the SIM phase shifts.

### 4.1. Convergence Behavior

**Figure 2:**
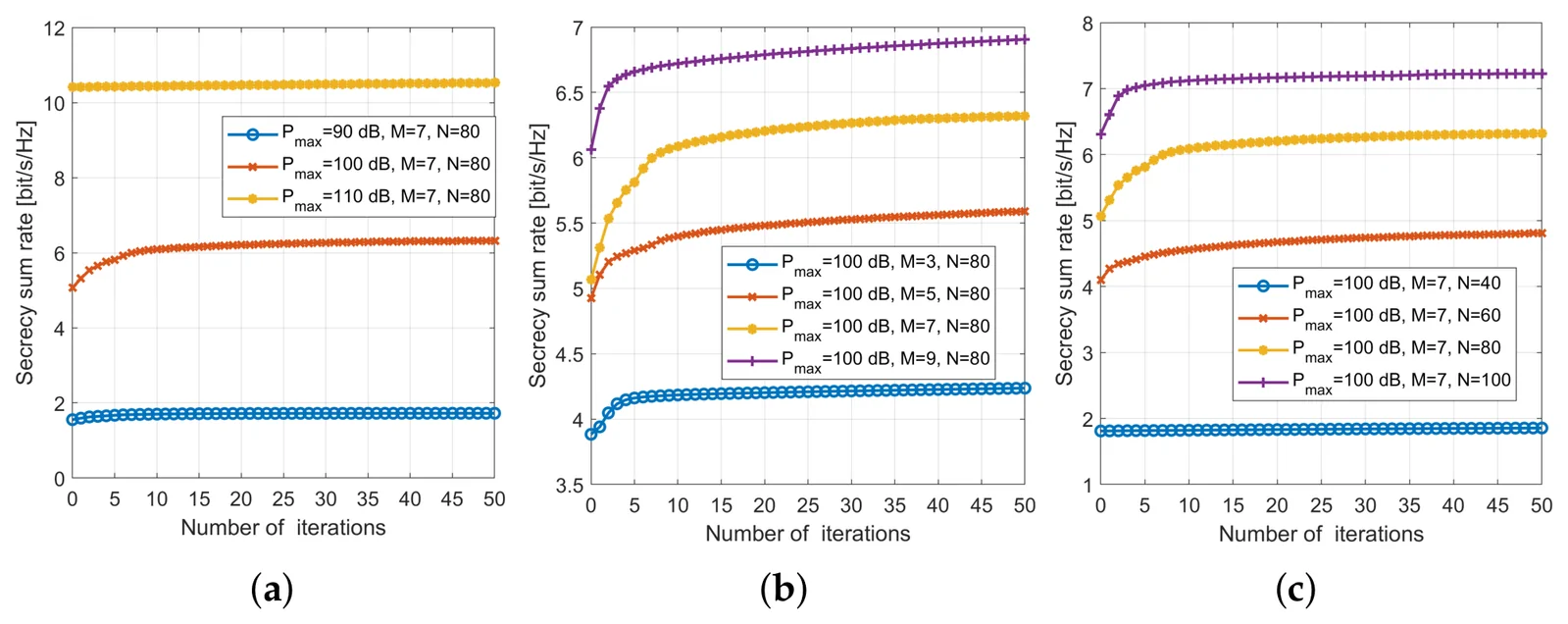
Convergence of the proposed MA–SIM algorithm with varying (**a**) transmit power, (**b**) number of MAs, and (**c**) number of SIM elements per layer.

Figure 2 depicts the convergence behavior over 50 outer iterations without early stopping. The maximum number of iterations for the beamforming, SIM phase, and MA position subproblems is also set to 50. For all tested configurations, the secrecy sum rate largely stabilizes within about 30 outer iterations, with only marginal improvements thereafter. The secrecy sum rate also increases with the transmit power, the number of MAs, and the number of elements per SIM layer.

### 4.2. Comparison with Benchmarks

**Figure 3:**
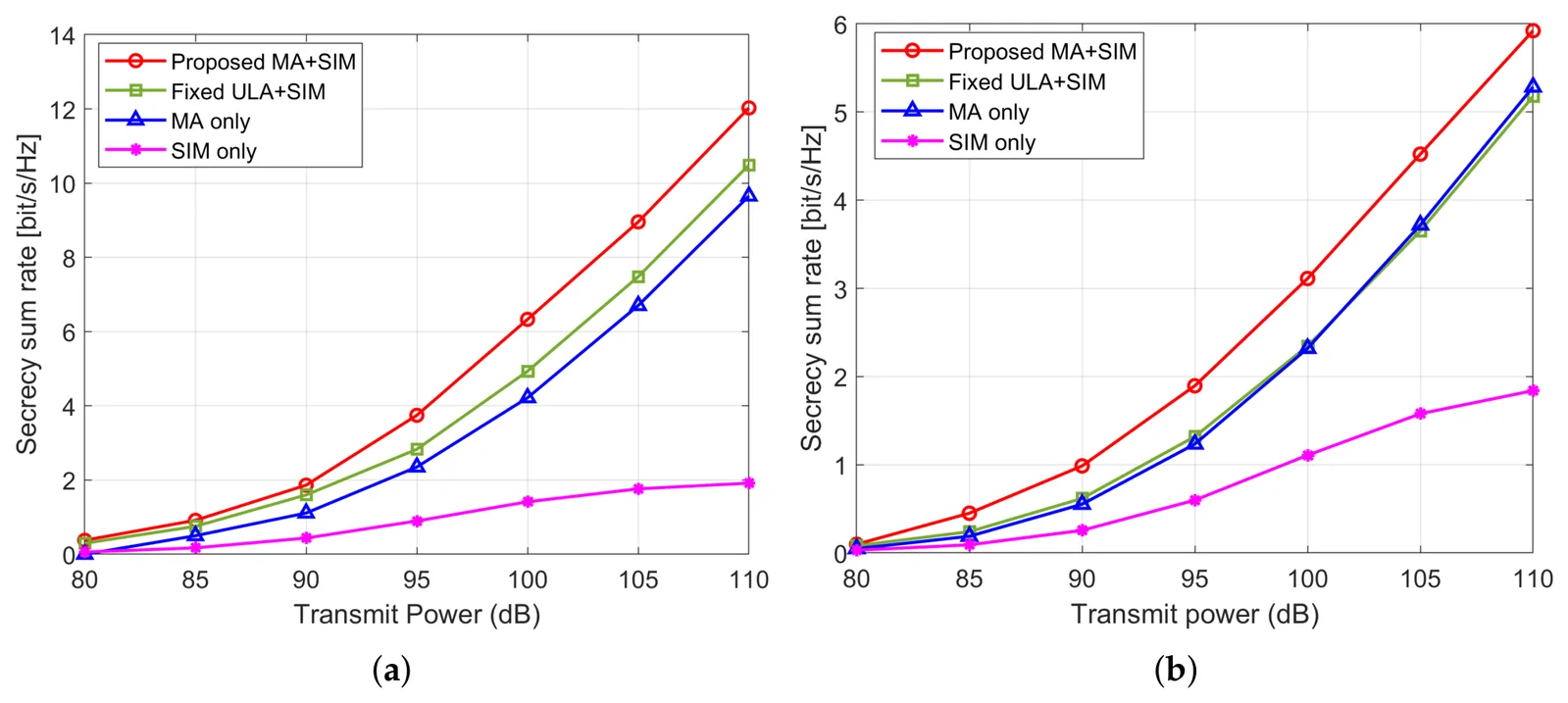
Secrecy sum-rate comparison versus normalized transmit power for (**a**) M=7 and N=80, and (**b**) M=3 and N=60.

Figure 3a compares the four schemes over *P* = 80:5:110 dB for the reference configuration M=7 and N=80. The proposed joint design achieves the largest secrecy sum rate at every tested power. At 100 dB, it attains 6.330 bit/s/Hz, compared with 4.932, 4.218, and 1.414 bit/s/Hz for Fixed ULA + SIM, MA only, and SIM only, respectively. The gain over the strongest benchmark, Fixed ULA + SIM, is therefore 28.3%. Figure 3b repeats the comparison with fewer spatial and wave-domain degrees of freedom, namely M=3 and N=60, while keeping K=J=2, L=2, and the remaining parameters unchanged. The proposed method again achieves the largest secrecy sum rate at every tested power. At 100 dB, the four rates are 3.112, 2.346, 2.320, and 1.108 bit/s/Hz for Proposed MA + SIM, Fixed ULA + SIM, MA only, and SIM only, respectively. The gain of the proposed scheme over the strongest benchmark is therefore 32.7%.

**Figure 4:**
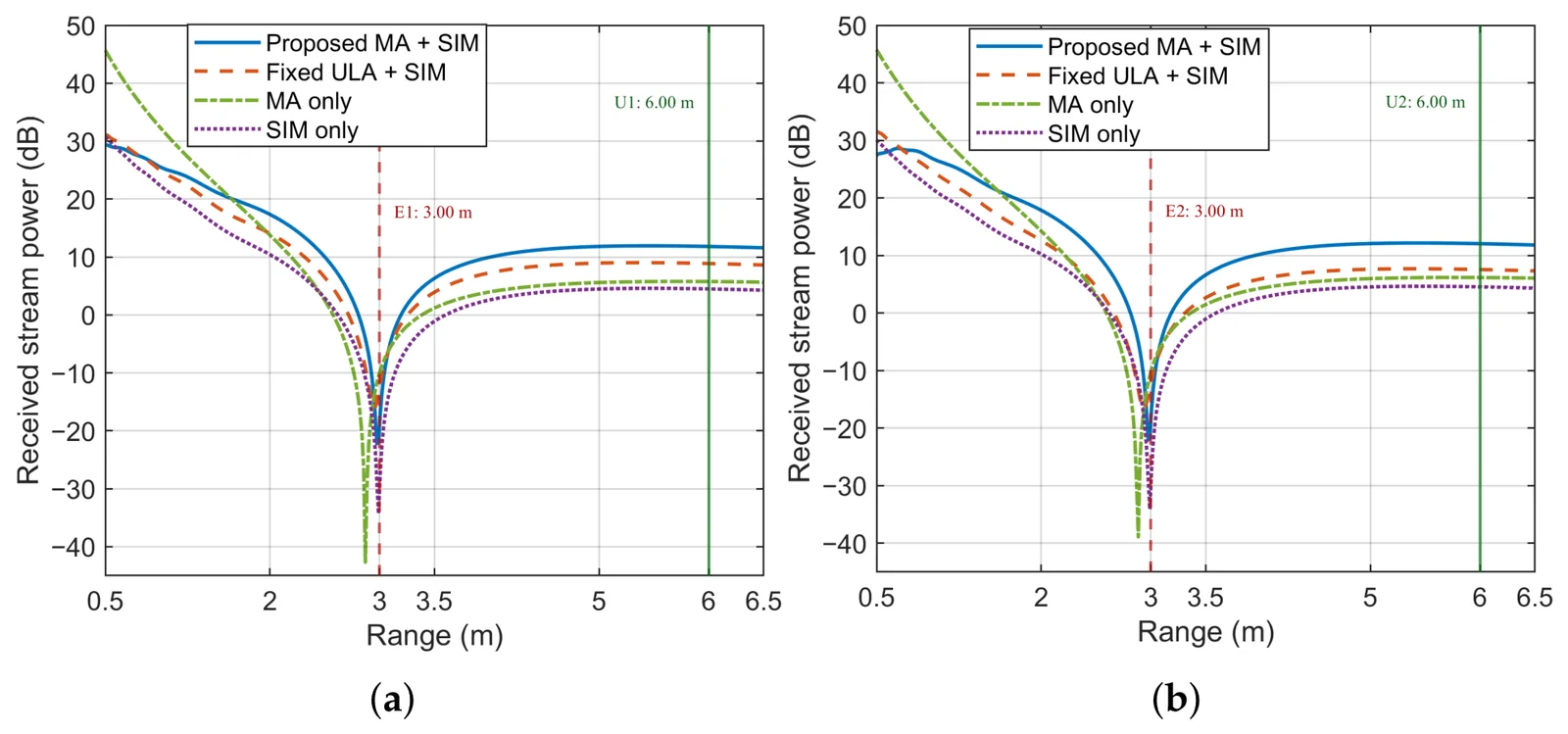
Absolute received power of the two confidential streams along radial directions: (**a**) stream 1 along the direction of user 1 ($-20^{\circ }$) and (**b**) stream 2 along the direction of user 2 ($17^{\circ }$).

To examine range selectivity directly, Figure 4 plots the absolute received power of each confidential stream along the direction of its intended user. For user direction $\vartheta_{k}$, the radial slice is defined as

(80) $$p_{k}^{\mathrm{rad}}\left(r;\vartheta_{k}\right)≜p_{k}\;\left({\left[\begin{array}{ccc} 0 & r\cos\vartheta_{k} & r\sin\vartheta_{k} \end{array}\right]}^{T}\right),$$

and the figure reports $10\log_{10}p_{k}^{\mathrm{rad}}\left(r;\vartheta_{k}\right)$. This absolute quantity retains the common USW path loss and therefore measures the combined effect of propagation and beam shaping rather than a normalized array factor. Here, we set P=100 dB, with M=7, N=100, K=J=2, and L=2. The paired Eve is located at r=3 m and the intended user at r=6 m along each direction, as marked by the red dashed and green solid lines. All four schemes use the same absolute dB scale.

As shown in Figure 4, all schemes form a sharp attenuation notch around the Eve range, but they retain different amounts of useful power at the intended user. At r=6 m, the proposed MA + SIM design delivers 11.81 and 12.04 dB for streams 1 and 2, respectively. Fixed ULA + SIM reaches 8.87 and 7.55 dB, MA only reaches 5.76 and 6.15 dB, and SIM only reaches 4.48 and 4.53 dB. Thus, relative to the strongest benchmark, the proposed design increases the intended-stream power by 2.94 dB for stream 1 and 4.49 dB for stream 2.

At the Eve range of 3 m, the proposed design suppresses the two stream powers to −17.24 and −16.95 dB, below the corresponding Fixed ULA + SIM values of −11.80 and −11.61 dB and the MA-only values of −9.65 and −9.86 dB. SIM only produces still deeper notches of approximately −27.6 dB, but it also yields the lowest intended-user power. Although the SIM-only scheme attains the deepest Eve notches, its secrecy performance is limited by weak useful signal power and inter-stream interference. With a single fixed transmit antenna and equal power allocation, both streams share the same spatial radiation pattern, giving each user equal desired and interfering power and thus an SINR below unity. The proposed method, by contrast, best balances leakage suppression and useful signal delivery.

### 4.3. Near-Field Versus Far-Field Optimization

**Figure 5:**
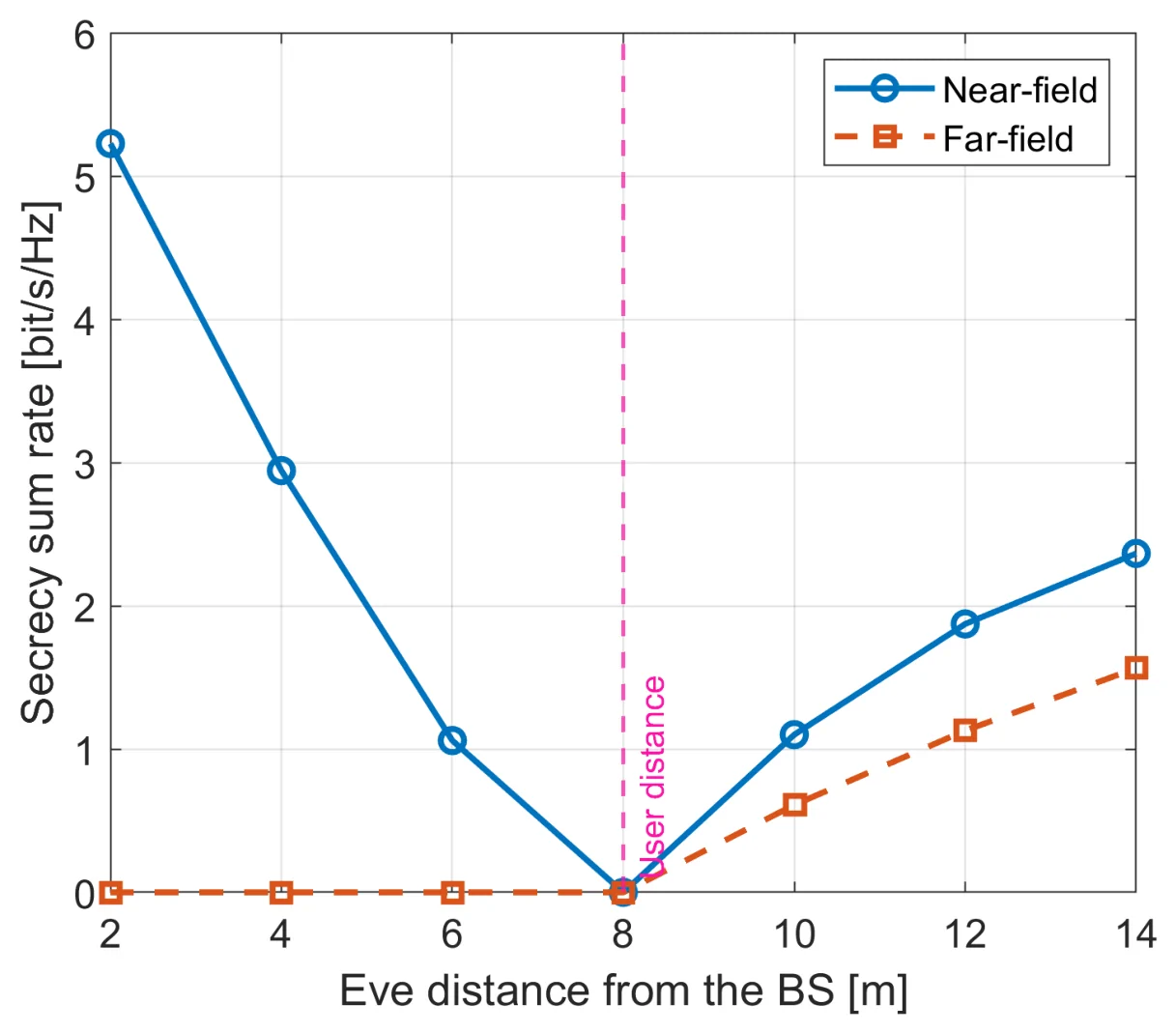
Near- and far-field MA–SIM optimization.

To evaluate the range discrimination capability in the near-field regime, we consider a scenario with K=J=1, while retaining M=7, N=80, L=2, and P=100 dB. The user is fixed at a range of 8 m and an angle of $10^{\circ }$, while Eve is placed at the same angle and moved from 2 to 14 m in 2 m increments. The SIM aperture is (N−1)λ/2=0.423 m, giving a Rayleigh distance of 33.43 m; therefore, all tested terminals lie within the radiative near-field region. The two curves in Figure 5 are obtained by independently optimizing the complete MA–SIM design under either the USW spherical-wave channel or a plane-wave far-field channel.

The far-field secrecy rate is zero when Eve is at 2, 4, 6, or 8 m. In the plane-wave model, receivers at the same angle have collinear normalized channel vectors. With equal noise powers, an Eve that is no farther than the user has at least the same channel gain, so no nonzero transmit state can produce a positive secrecy rate. By contrast, the near-field design achieves 5.227, 2.944, and 1.060 bit/s/Hz when Eve is at 2, 4, and 6 m, respectively. The near-field advantage arises from the distinct angle and distance dependence of the two channel models. In the far-field regime, the normalized spatial channel response depends only on the angle of departure. Thus, a user and an eavesdropper at the same angle have collinear channel vectors and cannot be spatially separated by any transmit beamformer. In the near-field regime, however, the spherical wavefront induces a distance dependent phase curvature, making the channel jointly dependent on angle and distance. This enables the proposed design to focus energy at the user distance while forming an attenuation notch at the eavesdropper distance, even without angular separation. Both models give zero secrecy rate when Eve and the user coincide at 8 m. When Eve is farther than the user, path loss also favors the legitimate link. However, the near-field design still achieves higher secrecy rates than the far-field design.

### 4.4. Impact of Imperfect CSI and Eve Location Error

**Figure 6:**
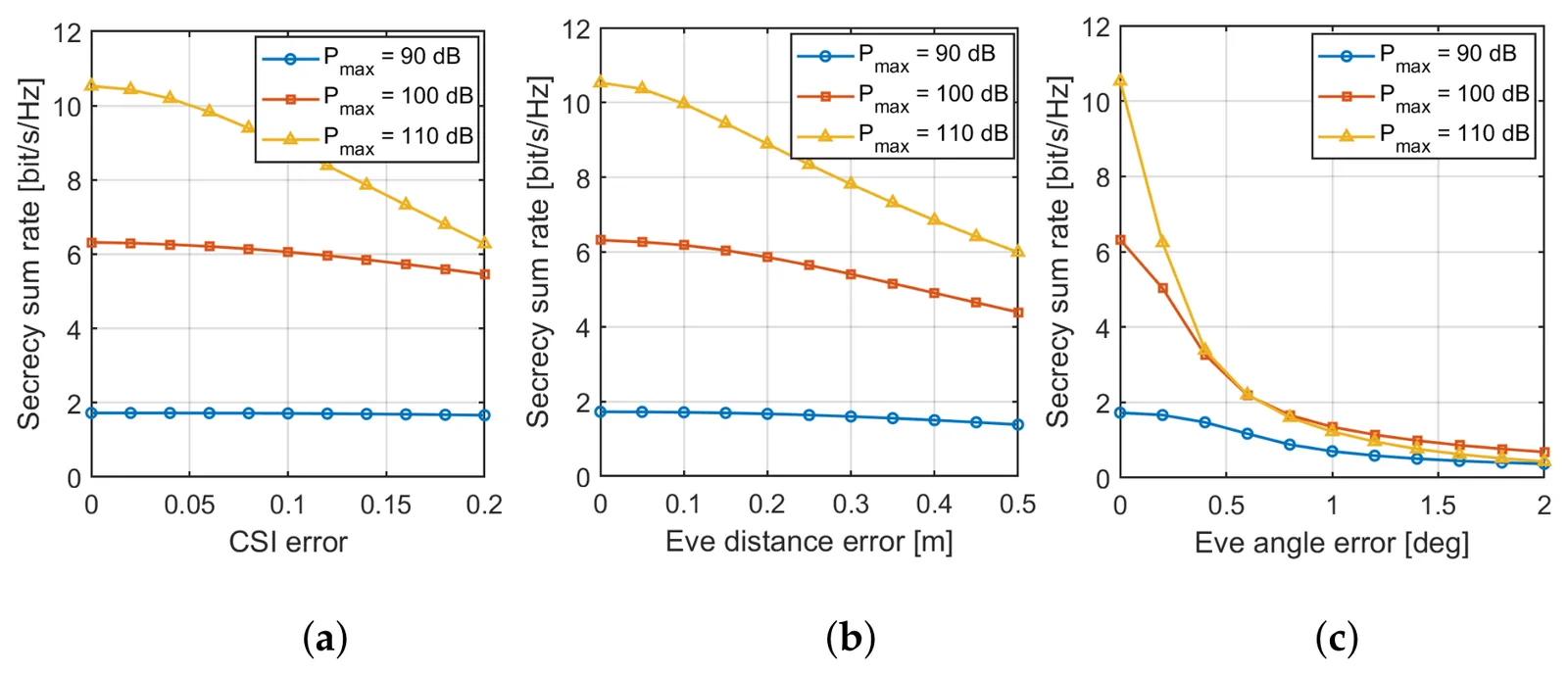
Sensitivity of the secrecy sum rate to (**a**) CSI error, (**b**) Eve range error, and (**c**) Eve angle error.

Since perfect channel state information (CSI) is generally unavailable in practice, we further conduct simulations to evaluate the proposed designs under separately applied CSI, Eve distance, and Eve angle uncertainties. For CSI mismatch, each actual last layer channel is modeled as $h=\hat{h}+e$, where $e∼\mathrm{CN}\;\left(0,\frac{\epsilon^{2}{∥\hat{h}∥}_{2}^{2}}{N}I_{N}\right)$ and the normalized mean-square error (NMSE) is $\mathrm{NMSE}=\frac{E\{∥e{∥}_{2}^{2}\}}{∥\hat{h}{∥}_{2}^{2}}$. The CSI error axis is the relative root mean-square (RMS) error $\sqrt{\mathrm{NMSE}}$, varied from 0 to 0.20. For Eve location uncertainty, we independently apply $\Delta r_{j}∼U\left[-a,a\right]$ and $\Delta \vartheta_{j}∼U\left[-b,b\right]$, with *a* measured in meters and *b* in degrees. The perturbed Eve channels are regenerated from the corresponding locations, while the user locations remain fixed. As shown in Figure 6, CSI mismatch and Eve distance uncertainty produce a gradual reduction in the secrecy sum rate, whereas Eve angle uncertainty causes a substantially sharper degradation. This contrast highlights the importance of accurate directional information for the algorithm design.

## 5. Conclusions

This paper investigates secure multi-user near-field transmission with MAs and a SIM in the presence of multiple eavesdroppers. We formulate a joint optimization problem that maximizes the secrecy sum rate by jointly designing the transmit beamformers, the MA positions, and the SIM phase shifts. To tackle this nonconvex problem, we develop an alternating optimization algorithm that decouples the original problem into three subproblems for transmit beamforming, SIM phase shifts, and MA positions, which are solved via WMMSE, a layer-by-layer method with relaxation and projection, and SCA, respectively. Simulation results demonstrate the effectiveness of the proposed joint design, showing consistent gains over benchmark schemes. The results provide numerical validation under the adopted channel models and idealized hardware assumptions, including sensitivity tests under CSI and Eve location uncertainty. Experimental validation using an MA–SIM prototype remains a direction for future research.

## Figures and Tables

**Table 1:** Comparison of related works.

Contributions	[2,5,6]	[7]	[10]	[11]	[18,19]	[20]	This Work
Near-field effect	✓	✓	×	×	×	✓	✓
SIM	×	×	✓	✓	×	×	✓
MAs	×	×	×	×	✓	✓	✓
Secrecy sum-rate objective	×	×	×	×	×	×	✓
Multiple eavesdroppers	×	✓	×	✓	×	×	✓

## Data Availability

Data are contained within the article. Further inquiries can be directed to the corresponding author.
